# Standalone Intra-Articular Injections for Temporomandibular Joint Disorders: Overview of Meta-Analytic Evidence

**DOI:** 10.3390/jcm15135208

**Published:** 2026-07-03

**Authors:** Wojciech Macek, Maciej Chęciński, Karolina Grzybowska-Kowalczyk, Maja Kosińska, Amelia Hoppe, Julia Kasprzycka, Oliwia Jagiełło, Tomasz Horodniczy, Zuzanna Baniak, Izabella Chyży, Kamila Chęcińska, Maciej Sikora

**Affiliations:** 1 Department of Oral Surgery, Preventive Medicine Center, Komorowskiego 12, 30-106 Cracow, Poland; ld.wojciech.macek@wp.pl (W.M.); majaakosinska@gmail.com (M.K.); amelia.a.hoppe@gmail.com (A.H.); 2 National Medical Institute of the Ministry of the Interior and Administration, Wołoska 137, 02-507 Warsaw, Poland; maciej.checinski@pimmswia.gov.pl (M.C.); ichyzy@gcm.pl (I.C.); maciej.sikora@pimmswia.gov.pl (M.S.); 3 Department of Maxillofacial Surgery, Hospital of the Ministry of the Interior and Administration, Wojska Polskiego 51, 25-375 Kielce, Poland; 4 Uśmiech Family Dental Clinic, Nastrojowa 26, 91-496 Łódź, Poland; karolina.orto.gk@gmail.com; 5 Miodowa Clinic, Głowaccy Medical and Dental Practice Professional Partnership, Kiekrz, Miodowa 2, 62-090 Rokietnica, Poland; 6 Gabinety Lekarskie Dent-im M.B. Pawelczyk Spółka Jawna, Różana 13/1, 61-577 Poznań, Poland; oliwiajagiellodent@gmail.com; 7 Ortho.pl Dental Office, Buforowa 34, 52-131 Wrocław, Poland; tomasz.horodniczy2@gmail.com; 8 Stomatologia Centrum, Moniuszki 3/7, 32-020 Wieliczka, Poland; baniakzuzanna@gmail.com; 9 Department of Biochemistry and Medical Chemistry, Pomeranian Medical University, Powstańców Wielkopolskich 72, 70-111 Szczecin, Poland

**Keywords:** temporomandibular joint disorders, intra-articular injections, platelet-rich plasma, hyaluronic acid, corticosteroids, osteoarthritis

## Abstract

**Background/Objectives**: Intra-articular injections are used for temporomandibular disorders (TMDs) resistant to conservative treatment. However, many reviews assess injectable agents combined with arthrocentesis or other co-interventions, limiting interpretation of their standalone effects. This overview aimed to summarize meta-analytic evidence on standalone intra-articular injections for temporomandibular joint disorders. **Methods**: MEDLINE, BASE, and Europe PMC were searched on 29 March 2026. Systematic reviews with quantitative meta-analyses evaluating standalone intra-articular TMJ injections were included. Data regarding injectable substances, clinical indications, and outcome domains were extracted and synthesized descriptively. **Results:** Three systematic reviews with meta-analyses were included. The evidence addressed platelet-rich plasma, corticosteroids, sodium hyaluronate, and physiological saline. Reported indications included degenerative joint disease, osteoarthritis, internal derangement, and arthritis. All included agents were reported to be associated with pain reduction. **Conclusions**: Meta-analytic evidence on standalone intra-articular injections for TMDs remains limited and heterogeneous. Available data suggest potential benefits, mainly for pain reduction, but do not establish clear superiority of any agent. The potential therapeutic activity of physiological saline should be considered when designing future injection trials.

## 1. Introduction

### 1.1. Rationale

Temporomandibular joint disorders (TMDs) constitute a heterogeneous group of conditions affecting the temporomandibular joint and associated structures, often presenting with pain, joint sounds, and functional limitations [1,2]. In cases where conservative management fails to provide adequate symptom relief, intra-articular injection therapies have emerged as widely applied minimally invasive treatment modalities [3,4]. These interventions involve the administration of various substances directly into the joint space, including hyaluronic acid, corticosteroids, platelet-rich plasma, and other biologically active agents [4,5,6]. Their proposed mechanisms include reduction in inflammation, improvement of joint lubrication, and promotion of tissue regeneration [4,6].

Although intra-articular injections are frequently administered following arthrocentesis, standalone injections may represent a less invasive therapeutic alternative in selected patients. Such approaches may be considered in cases of early degenerative joint disease, inflammatory joint disorders, or internal derangement where symptom control is desired without lavage procedures or surgical escalation. Nevertheless, the extent to which injectable substances exert clinically meaningful effects independently of arthrocentesis remains uncertain.

Over the past decade, a substantial number of randomized controlled trials have evaluated the effectiveness of intra-articular injections for TMDs, leading to the publication of multiple systematic reviews and meta-analyses [6,7]. However, the available evidence remains heterogeneous with respect to diagnostic classifications, injectable substances, treatment protocols, comparator interventions, and outcome measures [7,8]. This heterogeneity makes it difficult to obtain a clear and comprehensive overview of the current evidence landscape [7,8]. In addition, many reviews evaluate injectable agents together with arthrocentesis or other co-interventions, making interpretation of the independent effects of intra-articular substances difficult.

In this context, an overview of reviews may help provide a structured overview of which injectable substances, clinical indications, and outcome domains have already been quantitatively synthesized, while also identifying areas where evidence remains limited or absent [8].

### 1.2. Objectives

The objective of this review was to map the available meta-analytic evidence on standalone intra-articular injections for temporomandibular joint disorders. Specifically, the overview aimed to identify which injectable substances, clinical indications, and outcome domains have been quantitatively synthesized, and to determine where evidence remains limited or absent.

## 2. Materials and Methods

### 2.1. Protocol and Registration

This overview was performed and reported following the Preferred Reporting Items for Systematic Reviews and Meta-Analyses extension for Scoping Reviews checklist [9]. A review protocol was developed prior to study initiation and registered in the Open Science Framework repository under registration number: osf.io/ksmc4.

### 2.2. Eligibility Criteria

Eligibility criteria for this overview included systematic reviews containing quantitative meta-analyses that evaluated intra-articular injections for temporomandibular disorders (TMDs).

Standalone intra-articular injection was defined as administration of an injectable substance into the temporomandibular joint without concurrent arthrocentesis, lavage procedures, or additional intra-articular surgical interventions. Administration of local anesthesia solely for procedural comfort was not considered a co-intervention.

The population of interest comprised patients diagnosed with TMJ-related conditions, including internal derangement or disc displacement, osteoarthritis or degenerative joint disease, arthralgia, and other painful TMJ-related disorders, provided that the intervention targeted the temporomandibular joint. No age restrictions were applied.

Eligible interventions were standalone intra-articular injections into the TMJ, irrespective of the injectable agent, dose, formulation, or number of administrations. Reviews were eligible regardless of the comparator used in the source trials, provided that the effect of standalone intra-articular injection therapy could be identified. Eligible comparators could therefore include placebo or sham procedures, no treatment, conservative care, another injectable agent, or procedural interventions such as arthrocentesis. Reviews in which injectable agents were evaluated only as adjuncts to arthrocentesis, arthroscopy, surgery, or broader multimodal treatment, without separate reporting of standalone injection effects, were excluded.

Outcomes of interest included pain intensity, mandibular function, maximum mouth opening, patient-reported outcomes, and adverse events when reported.

The review focused on systematic reviews with quantitative meta-analyses, including standalone meta-analyses and reviews containing a meta-analytic component. No restrictions were applied regarding publication date or language. Non-English reports were eligible if the required data could be extracted or obtained through translation. Narrative reviews without quantitative pooling, case reports, case series, animal studies, in vitro studies, editorials, and conference abstracts without full meta-analytic data were excluded. Because this overview used systematic reviews as the unit of analysis and only three eligible meta-analyses were identified, subgroup and sensitivity analyses were not methodologically appropriate; sources of heterogeneity were therefore explored descriptively. Eligibility criteria are summarized in Table 1.

### 2.3. Information Sources

Searches were conducted on 29 March 2026, in three freely accessible electronic sources: MEDLINE via PubMed, Bielefeld Academic Search Engine (BASE), and Europe PMC. These sources were selected to ensure that the search strategy could be independently reproduced without reliance on subscription-only databases.

PubMed is a free resource developed and maintained by the National Center for Biotechnology Information at the U.S. National Library of Medicine, National Institutes of Health, and contains more than 40 million citations and abstracts of biomedical and life sciences literature [10]. BASE is a multidisciplinary academic search engine operated by Bielefeld University Library, indexing more than 400 million records from more than 12,000 content providers, including journals, institutional repositories, and digital collections [11]. Europe PMC provides free access to life sciences literature from trusted sources, including journal articles, preprints, books, reviews, protocols, and other documents enriched with links to supporting data and related resources [12].

### 2.4. Search

Searches for systematic reviews and meta-analyses were conducted in medical databases. The query was formulated based on the eligibility criteria specified above. The following query was finally used in all search engines: (“temporomandibular” OR “TMJ” OR “TMD”) AND (“intra-articular” OR “injection” OR “hyaluronan” OR “PRP” OR “corticosteroid” OR “botulinum” OR “local anesthetic” OR “autologous blood” OR “stem-cell preparations” OR “growth factors”) AND (“systematic review” OR “meta-analysis”).

### 2.5. Selection of Sources

All records retrieved from the database searches were imported into Rayyan (web application; Rayyan Systems Inc., Cambridge, MA, USA), where duplicates were removed by one reviewer (T.H.) [13]. Screening was conducted in two stages. Before formal screening, reviewers performed a calibration exercise to ensure consistent application of the eligibility criteria. In the first stage, titles and abstracts were independently and blindly screened by two reviewers (J.K. and O.J.) to identify potentially eligible reports. In the second stage, full-text reports were independently assessed against the eligibility criteria by the same reviewers. Disagreements were resolved through discussion and consensus. In case of further disagreement between the two primary researchers, a third researcher was supposed to be added to resolve such a problem. Such a situation never presented itself. Inter-rater agreement was assessed using Cohen’s kappa coefficient for nominal decisions, calculated separately for the title/abstract screening stage and the full-text assessment stage based on reviewers’ independent decisions before consensus resolution [14]. Reasons for exclusion at the full-text stage were documented. The study selection process is presented in the PRISMA flow diagram generated using the PRISMA2020 Shiny application (PRISMA2020 v1.1.3; Evidence Synthesis Hackathon, hosted via shinyapps.io) [15].

### 2.6. Data Charting Process

Data from the included studies were extracted independently by two reviewers (J.K. and O.J.) using predefined tables prepared in Google Sheets. Extracted data were compared between reviewers to ensure consistency and accuracy. Discrepancies were resolved through discussion and consensus.

### 2.7. Data Items

Extracted data included injectable substances, clinical indications, comparator characteristics, and reported outcome domains.

### 2.8. Critical Appraisal of Individual Sources of Evidence

The methodological quality of the included meta-analyses was assessed using the Joanna Briggs Institute Critical Appraisal Checklist for Systematic Reviews and Research Syntheses to provide general methodological context for the included evidence [16].

### 2.9. Synthesis of Results

Results were synthesized descriptively and grouped according to injectable substance, clinical indication, comparator characteristics, and outcome domain. Findings were summarized narratively and presented in tabular form. No additional quantitative synthesis was performed.

## 3. Results

### 3.1. Selection of Sources of Evidence

Three systematic reviews with meta-analyses met the eligibility criteria and were included in this overview.

**Figure 1:**
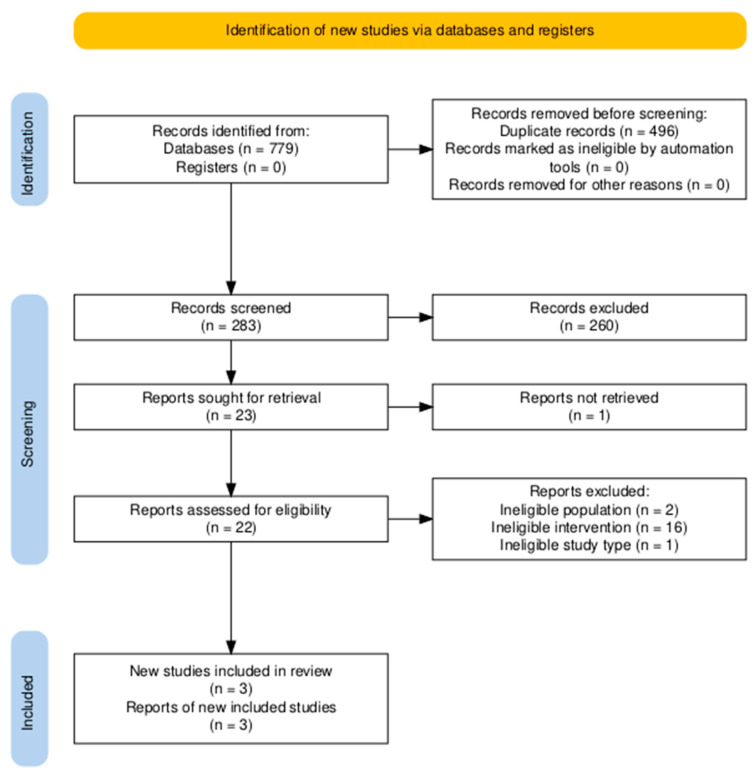
PRISMA flow diagram [15].

Inter-rater agreement was high at both screening stages, with Cohen’s κ = 0.95 for title and abstract screening and κ = 1.00 for full-text assessment. Disagreements were resolved by consensus. Reasons for exclusion at the full-text stage are presented in Table A1, and the complete selection process is shown in the PRISMA flow diagram (Figure 1).

### 3.2. Characteristics of Sources of Evidence

Three systematic reviews with meta-analyses published between 2018 and 2026 were included in this overview. All included reviews synthesized evidence from randomized controlled trials evaluating standalone intra-articular injectable therapies for temporomandibular joint disorders [17,18,19].

The included reviews addressed internal derangement, osteoarthritis, arthritis, degenerative joint disease, and temporomandibular joint degenerative disease. Investigated injectable agents included platelet-rich plasma, corticosteroids, sodium hyaluronate, and physiological saline. Comparator interventions varied across reviews and included placebo or saline control groups, other injectable agents, hyaluronic acid, corticosteroids, and procedural interventions such as arthrocentesis [17,18,19].

The number of randomized controlled trials included in the reviews ranged from 6 to 7, although not all studies were quantitatively synthesized in every meta-analysis. The main outcome domains were pain intensity and mandibular function, particularly maximum mouth opening. Some reviews additionally evaluated patient-reported outcomes, joint sounds, and adverse effects [17,18,19].

Overall, the included reviews reported that intra-articular injectable therapies were associated mainly with pain reduction in temporomandibular joint disorders [17,18,19]. One review additionally reported improvement in mandibular function following platelet-rich plasma injections [17].

### 3.3. Critical Appraisal Within Sources of Evidence

The included meta-analyses generally met most Joanna Briggs Institute checklist criteria. The main recurring limitation was unclear assessment of publication bias across all included reviews. Their strengths include a clear formulation of the review question, the definition of appropriate inclusion criteria, and the use of appropriate statistical methods for data synthesis. Additionally, most reviews included a critical appraisal of the primary studies.

The primary studies synthesized in these meta-analyses had relatively small sample sizes (often <50 participants per group), short follow-up periods (usually ≤6 months), and insufficient blinding of participants and personnel. Item-level JBI critical appraisal results for each included review are presented in Table 2.

### 3.4. Results of Individual Sources of Evidence

The characteristics and main findings of the included meta-analyses are summarized in Table 3 and further numerical results are presented in Table 4.

Wielandt et al. (2026) [17] assessed the efficacy and safety of platelet-rich plasma injections administered without arthrocentesis in patients with temporomandibular joint degenerative disease. The review synthesized evidence from six randomized controlled trials and reported improvements in both pain and mandibular function following intra-articular PRP injections. The observed effects were considered comparable to arthrocentesis-based interventions, although the certainty of evidence was described as low to moderate [17].

Chęciński et al. (2024) [19] investigated the effects of intra-articular physiological saline injections in patients with internal derangement, arthritis, or degenerative joint disease of the temporomandibular joint. Based on seven randomized controlled trials, the meta-analysis demonstrated that saline injections were associated with pain reduction. The review therefore questioned the assumption that physiological saline acts as a completely inert placebo in temporomandibular joint injection studies [19].

Moldez et al. (2018) [18] evaluated intra-articular sodium hyaluronate and corticosteroid injections in patients with osteoarthritis and internal derangement of the temporomandibular joint. The review included seven randomized controlled trials, although only four studies were incorporated into the quantitative synthesis. Both injectable agents were associated with pain reduction, and pooled analyses did not demonstrate statistically significant differences between sodium hyaluronate and corticosteroids. Outcome domains primarily included pain intensity and mandibular function [18].

Overall, the included meta-analyses suggested that standalone intra-articular injectable therapies may be associated with beneficial clinical outcomes in temporomandibular joint disorders, particularly pain reduction. However, interpretation of the findings remains limited by heterogeneity in clinical indications, injectable agents, comparator interventions, outcome domains, and methodological characteristics of the included primary studies [17,18,19].

### 3.5. Synthesis of Results

The available meta-analytic evidence was concentrated mainly in internal derangement and degenerative joint disease of the temporomandibular joint (Table 5). Pain intensity was the most consistently evaluated outcome domain across all included reviews, whereas mandibular function and maximum mouth opening were assessed less frequently. Evidence regarding joint sounds, adverse effects, and patient-reported outcomes was limited.

## 4. Discussion

### 4.1. Summary of Evidence

The present overview indicates that meta-analytic evidence on standalone intra-articular injections for temporomandibular joint disorders remains limited and heterogeneous. The available evidence is concentrated primarily on pain-related outcomes, whereas mandibular function and other clinically relevant domains have been evaluated less consistently. Across the included meta-analyses, platelet-rich plasma, corticosteroids, sodium hyaluronate, and physiological saline were generally associated with clinical improvement, particularly with respect to pain reduction, although the certainty and consistency of evidence varied across interventions and clinical indications.

The identified evidence also highlights substantial heterogeneity in diagnostic classifications, injection protocols, comparator interventions, and outcome reporting. This heterogeneity complicates direct comparison between injectable agents and limits the interpretability of pooled estimates. In particular, many published reviews in this field combine intra-articular injections with arthrocentesis or other co-interventions, making it difficult to isolate the independent effects of injectable substances. The present evidence map specifically focused on standalone intra-articular injections in an attempt to address this methodological issue.

Among the included reviews, platelet-rich plasma was associated with improvements in both pain and mandibular function in patients with temporomandibular joint degenerative disease, although the certainty of evidence was reported as low to moderate [17]. Corticosteroids and sodium hyaluronate were both associated with pain reduction in osteoarthritis and internal derangement, without clear evidence of superiority of either agent [18]. Additionally, the findings regarding physiological saline suggest that saline injections may exert therapeutic effects rather than functioning as a completely inert placebo [11]. The observed effects associated with physiological saline may reflect a combination of procedural effects, joint distension, lavage-like mechanisms, placebo responses, or biological effects of the injected solution itself. Consequently, saline should not necessarily be regarded as a completely inert control intervention. This observation may have important implications for the interpretation and design of future injection trials in temporomandibular joint disorders.

Since publication of the included meta-analyses, additional randomized clinical trials have continued to evaluate intra-articular injectable therapies for TMDs. Although these studies do not fundamentally alter the conclusions of the present overview, they illustrate the continuing development of the field and highlight the need for updated evidence syntheses.

Taken together, the currently available evidence suggests that standalone intra-articular injections may provide clinical benefit in selected temporomandibular joint disorders, particularly for pain-related outcomes. However, the limited number of eligible meta-analyses, heterogeneity of the underlying randomized controlled trials, and variability in certainty of evidence preclude firm conclusions regarding comparative effectiveness, superiority between injectable agents, or long-term treatment outcomes. These findings should not be interpreted as representing the entire clinical literature on TMJ injection therapy, but rather the currently available meta-analytic evidence on standalone intra-articular injections meeting strict eligibility criteria.

### 4.2. Safety Considerations

Safety interpretation should account for both the pharmacological properties of injectable agents and the procedural aspects of intra-articular administration. The agents identified in the included meta-analyses differ substantially in their biological activity. Platelet-rich plasma is an autologous biologically active preparation, corticosteroids primarily exert anti-inflammatory effects, sodium hyaluronate is used to improve joint lubrication and modulate inflammatory processes, whereas physiological saline is usually treated as a control intervention despite possible clinical effects [19,20].

Although direct safety evidence from temporomandibular joint injection studies remains limited, broader injection-based literature provides useful contextual information. Hyaluronic acid has generally been reported as well tolerated in non-TMJ local injection applications, and systematic reviews from other clinical areas have described a favorable safety profile for hyaluronic acid-based interventions [20,21]. However, extrapolation of these findings to temporomandibular joint injections should be cautious because anatomical conditions, injection technique, dose, and indication differ across applications.

Across the included evidence, serious adverse events were not prominently reported; however, safety data were limited and inconsistently presented [17,18,19]. The apparent safety of standalone intra-articular injections should therefore be interpreted cautiously, particularly because the underlying randomized controlled trials were generally small and had relatively short follow-up periods.

### 4.3. Future Research Directions

Future research should aim to improve methodological consistency in studies of temporomandibular joint injections, particularly through standardization of diagnostic criteria, intervention protocols, comparator selection, follow-up duration, and outcome measures. Alignment with established diagnostic frameworks would improve comparability across studies [1]. Current evidence remains characterized by variability in study design, interventions, comparators, and follow-up periods, as highlighted in recent reviews and meta-analyses [6,7]. Moreover, future trials and reviews should use standardized core outcome domains, consistently report functional outcomes and adverse events, and include longer follow-up periods.

There is a need for well-designed randomized controlled trials directly comparing platelet-rich plasma, hyaluronic acid or sodium hyaluronate, corticosteroids, physiological saline, and other injectable agents within clearly defined temporomandibular joint disorder subgroups. Future studies should clearly distinguish standalone intra-articular injections from injections used as adjuncts to arthrocentesis, arthroscopy, or other multimodal interventions. This distinction is essential for determining whether observed clinical effects are attributable to the injectable substance, the intra-articular procedure itself, or accompanying interventions.

Comparator selection should also be carefully reconsidered. Placebo or sham-controlled designs may help clarify substance-specific effects, but physiological saline may not always function as a fully inert comparator in temporomandibular joint injection studies [19]. At present, part of the comparative understanding of injectable agents is derived from studies conducted in other joints, including knee and hip osteoarthritis [22,23,24]. Such evidence may provide useful context, but direct extrapolation to temporomandibular joint disorders should be approached cautiously because joint anatomy, biomechanics, disease mechanisms, and injection protocols differ substantially.

### 4.4. Strengths

This overview provides an updated and structured overview of meta-analytic evidence on standalone intra-articular injections for temporomandibular joint disorders. By focusing on reviews in which standalone injection effects could be identified, it addresses an important methodological issue in this field: the frequent combination of injectable agents with arthrocentesis, arthroscopy, or other co-interventions.

The review was conducted using predefined eligibility criteria, a structured search across multiple databases, independent and blinded screening, and duplicate data extraction. Reviewer calibration and assessment of inter-rater agreement further supported the consistency of the selection process.

Another strength is the organization of evidence according to injectable substance, clinical indication, comparator characteristics, and outcome domain. This approach allows areas with existing quantitative synthesis, such as pain-related outcomes, to be distinguished from domains where meta-analytic evidence remains limited or absent.

The absence of date and language restrictions also broadened the scope of evidence capture, although practical limitations related to accessibility and translation should still be considered.

### 4.5. Limitations

Several limitations should be considered. First, this overview was intentionally restricted to systematic reviews containing quantitative meta-analyses. As a result, relevant primary studies, emerging clinical evidence, and reviews without quantitative synthesis were not included.

Second, the included meta-analyses differed in study populations, diagnostic criteria, injectable agents, injection protocols, comparator interventions, follow-up periods, and outcome reporting. This heterogeneity limits direct comparison between interventions and precludes integrative conclusions regarding the relative effectiveness of individual injectable agents.

Third, overlap of primary studies across the included meta-analyses may have occurred, potentially leading to partial duplication of evidence. In addition, although no publication date or language restrictions were applied, relevant evidence may have been missed because only selected databases were searched and practical limitations related to full-text accessibility or translation may have affected study inclusion. Although the use of freely accessible databases strengthened reproducibility, the absence of subscription-based sources may have introduced a risk of database selection bias.

Finally, this review was designed as a descriptive evidence map. No additional quantitative synthesis, formal certainty assessment, or re-analysis of primary trial data was performed. The findings of this overview should be interpreted in light of several limitations. Only three eligible meta-analyses were identified, limiting the breadth of available evidence. Furthermore, the included reviews differed substantially with respect to diagnostic criteria, injectable substances, comparator interventions, outcome measures, and follow-up duration. These factors limit direct comparison across studies and reduce confidence in comparative conclusions.

## 5. Conclusions

This overview identified three meta-analyses evaluating standalone intra-articular injections for temporomandibular joint disorders. The available meta-analytic evidence addressed platelet-rich plasma, corticosteroids, sodium hyaluronate, and physiological saline in patients with degenerative joint disease, osteoarthritis, internal derangement, and arthritis.

Pain intensity was the most consistently assessed outcome across the included reviews. All evaluated injectable agents were reported to be associated with pain reduction in at least one clinical indication, whereas evidence regarding mandibular function, joint sounds, patient-reported outcomes, safety, and long-term outcomes was limited. No clear superiority of any injectable agent was demonstrated based on the available meta-analytic evidence.

Overall, the evidence base remains limited, with only a small number of eligible meta-analyses and substantial heterogeneity in patient populations, interventions, comparators, and outcome measures. Evidence was concentrated mainly on pain-related outcomes, while several clinically relevant outcome domains remain insufficiently synthesized.

## Figures and Tables

**Table 1:** Eligibility criteria.

Category	Inclusion Criteria	Exclusion Criteria
Study type	Systematic reviews with quantitative meta-analysis	Reviews without quantitative pooling; non-review publications
Population	Patients with TMJ-related disorders	Non-human studies; non-TMJ conditions
Intervention	Standalone intra-articular TMJ injections	Adjunctive or multimodal interventions without separable standalone injection effects
Comparator	Any comparator	Effects not separable
Outcomes	Pain, mandibular function, patient-reported outcomes, adverse events	No relevant clinical outcomes
Timeframe/language	No date or language restrictions	Data not extractable or not translatable

Abbreviations: TMJ, temporomandibular joint.

**Table 2:** Joanna Briggs Institute critical appraisal of included systematic reviews with meta-analyses.

Question	Wielandt et al., 2026 [17]	Chęciński et al., 2024 [19]	Moldez et al., 2018 [18]
Is the review question clearly and explicitly stated?	Yes	Yes	Yes
Were the inclusion criteria appropriate for the review question?	Yes	Yes	Yes
Was the search strategy appropriate?	Yes	Yes	Yes
Were the sources and resources used to search for studies adequate?	Yes	Yes	Yes
Were the criteria for appraising studies appropriate?	Yes	Yes	Yes
Was critical appraisal conducted by two or more reviewers independently?	Yes	Yes	Yes
Were there methods to minimize errors in data extraction?	Yes	Yes	Yes
Were the methods used to combine studies appropriate?	Yes	Yes	Yes
Was the likelihood of publication bias assessed?	Unclear	Unclear	Unclear
Were recommendations for policy and/or practice supported by the reported data?	Yes	Unclear	Yes
Were the specific directives for new research appropriate?	Yes	Yes	Yes

“Unclear” indicates that the criterion could not be confidently assessed based on the information reported in the review.

**Table 3:** Characteristics of included meta-analyses on standalone intra-articular injections for temporomandibular joint disorders.

Author, Year	Duration of Effect	Authors’ Conclusion
Wielandt et al., 2026 [17]	No clear superior analgesic effect up to 6 months	PRP showed little to no difference compared with arthrocentesis for pain reduction.
Wielandt et al., 2026 [17]	Improvement observed up to 6 months	PRP may improve mandibular opening compared with arthrocentesis, although the certainty of evidence was low and the authors interpreted the effect cautiously.
Wielandt et al., 2026 [17]	No clear effect up to 6 months	Evidence was very uncertain regarding the effect of PRP on TMJ joint sounds.
Wielandt et al., 2026 [17]	Difference observed at 6 months, but based on one small RCT	Based on one small RCT, arthrocentesis + PRP showed lower pain than PRP alone at 6 months; however, the authors concluded that overall differences were limited and should be interpreted with caution.
Wielandt et al., 2026 [17]	No clear superior effect up to 6 months	PRP alone and arthrocentesis + PRP showed little to no difference in MMO.
Wielandt et al., 2026 [17]	Possible benefit up to 3 months, but not statistically significant	PRP may provide greater pain relief than HA at 3 months, although the evidence was limited and uncertain.
Wielandt et al., 2026 [17]	Possible reduction up to 3 months, but not statistically significant	PRP may reduce TMJ sounds compared with HA, but the evidence was uncertain.
Moldez et al., 2018 [18]	No comparative superiority demonstrated in the short term	NaH and CS did not differ significantly in short-term pain reduction.
Moldez et al., 2018 [18]	No comparative superiority demonstrated up to 1 year	NaH and CS did not differ significantly in long-term pain reduction.
Moldez et al., 2018 [18]	No comparative superiority demonstrated up to 2 years	The proportion of patients reporting symptom improvement was similar between NaH and CS.
Moldez et al., 2018 [18]	Effect demonstrated up to 6 months	NaH was superior to placebo in the number of patients reporting symptom improvement.
Chęciński et al., 2024 [19]	Significant pain reduction observed after 1 week	Intra-articular physiological saline was associated with significant short-term reduction in TMJ pain.
Chęciński et al., 2024 [19]	Significant pain reduction observed up to approximately 1 month	Intra-articular physiological saline was associated with significant reduction in TMJ pain at 4–6 weeks.
Chęciński et al., 2024 [19]	Pain remained below baseline up to 24 weeks, but without pooled CI or *p*-value	A weak but stable pain-reducing effect of intra-articular physiological saline was observed, although the mechanism remains uncertain.

Abbreviations: PRP—platelet-rich plasma; TMJ—temporomandibular joint; RCT—randomized controlled trial; MMO—maximum mouth opening; HA—hyaluronic acid; NaH—sodium hyaluronate; CS—corticosteroids; CI—confidence interval; *p*-value—probability value. Follow-up durations are reported as stated in the original studies.

**Table 4:** Numerical results presented in included studies.

Author, Year	Diagnosis	Injectable Agent	Comparator	Outcome	Effect Estimate	95% CI	*p* Value	Follow-Up in Months
Wielandt et al., 2026 [17]	TMJ DJD/OA	PRP	Arthrocentesis	Pain	MD = −1.12	−2.38 to 0.14	0.08	6
Wielandt et al., 2026 [17]	TMJ DJD/OA	PRP	Arthrocentesis	MMO	MD = 2.52 mm	0.68 to 4.36	0.007 *	6
Wielandt et al., 2026 [17]	TMJ DJD/OA	PRP	Arthrocentesis	Sounds	RR = 0.71	0.36 to 1.36	0.30	6
Wielandt et al., 2026 [17]	TMJ DJD/OA	PRP	Arthrocentesis + PRP	Pain	MD = 1.45	0.58 to 2.32	not reported	6
Wielandt et al., 2026 [17]	TMJ DJD/OA	PRP	Arthrocentesis + PRP	MMO	MD = −3.18 mm	−8.20 to 1.84	not reported	6
Wielandt et al., 2026 [17]	TMJ DJD/OA	PRP	HA	Pain	SMD = −1.84	−4.18 to 0.50	0.12	3
Wielandt et al., 2026 [17]	TMJ DJD/OA	PRP	HA	Sounds	RR = 0.27	0.04 to 2.02	0.20	3
Moldez et al., 2018 [18]	TMJ OA and/or ID	NaH	CS	Pain	SDM = −0.040	−0.395 to 0.315	0.825	1–1.5
Moldez et al., 2018 [18]	TMJ OA and/or ID	NaH	CS	Pain	SDM = −0.058	−1.055 to 0.939	0.909	6–12
Moldez et al., 2018 [18]	TMJ OA and/or ID	NaH	CS	Symptom improvement	RR = 0.994	0.540 to 1.830	0.985	1, 12, and 24 combined
Moldez et al., 2018 [18]	TMJ OA and/or ID	NaH	Placebo	Symptom improvement	RR = 3.194	1.357 to 7.518	0.008 *	1 and 6 combined
Chęciński et al., 2024 [19]	TMJ ID, arthritis or degeneration	NS	Baseline pain value/pre-injection value	Articular pain	Pain reduction = −23.72%; SE = 0.84%	−24.38% to −21.06%	<0.01 *	0.25
Chęciński et al., 2024 [19]	TMJ ID, arthritis or degeneration	NS	Baseline pain value/pre-injection value	Articular pain	Pain reduction = −34.01%; SE = 1.09%	−36.16% to −31.86%	<0.01 *	1–1.5
Chęciński et al., 2024 [19]	TMJ ID, arthritis or degeneration	NS	Baseline pain value/pre-injection value	Articular pain	Pain-to-baseline ratio: 73.3% at 12 weeks and 76.0% at 24 weeks	Not reported	Not reported	3–6

Abbreviations: DJD—degenerative joint disease; ID—internal derangement; OA—osteoarthritis; TMJ—temporomandibular joint; PRP—Platelet-rich plasma; NaH—Sodium hyaluronate; NS—normal saline/0.9% NaCl/physiological saline; HA—Hyaluronic acid; CS—Corticosteroids; MMO—Maximum mouth opening; Sounds—TMJ joint sounds; Pain—pain intensity as defined and assessed in the original study, including visual analog scale assessment where specified; Articular pain—articular pain intensity as defined and assessed in the original study Symptom improvement—patient-reported improvement of symptoms; CI—confidence interval; MD—mean difference; RR—risk ratio; SDM—standardized difference in means; SE—standard error; SMD—standardized mean difference; * Statistically significant; Follow-up values are reported in months. Weeks were converted to months using 4 weeks = 1 month; 1 year was converted to 12 months.

**Table 5:** Distribution of available meta-analytic evidence on standalone intra-articular injections across clinical indications and outcome domains.

Clinical Indication	Injectable Substance(s)	Outcome Domains Assessed	Included Review(s)
Internal derangement	Physiological saline; corticosteroids; sodium hyaluronate	Pain; mandibular function	Chęciński et al., 2024 [19]; Moldez et al., 2018 [18]
Arthritis	Physiological saline	Pain	Chęciński et al., 2024 [19]
Degenerative joint disease	Platelet-rich plasma; physiological saline; corticosteroids; sodium hyaluronate	Pain; mandibular function; safety-related outcomes	Wielandt et al., 2026 [17]; Chęciński et al., 2024 [19]; Moldez et al., 2018 [18]

Note: In the included meta-analyses, the term “internal derangement” was not consistently subdivided into disc displacement with reduction and disc displacement without reduction to avoid unsupported reclassification. The meta-analysis by Chęciński et al. (2024) [19] included mixed study populations overlapping mainly with internal derangement and degenerative joint disease rather than a standalone arthritis population.

## Data Availability

Data is contained within the article.

## Abbreviations

The following abbreviations are used in this manuscript:

BASE	Bielefeld Academic Search Engine
PRP	Platelet-rich plasma
RCT	Randomized controlled trial
TMDs	Temporomandibular disorders
TMJ	Temporomandibular joint

## Appendix A

**Table A1:** Studies excluded after full-text assessment.

Title	First Author, Publication Year	Reason for Exclusion
Efficacy and Safety of Intra-Articular Therapies for Temporomandibular Disorders: A Systematic Review and Meta-Analysis	Dos Santos et al., 2026 [25]	Ineligible intervention (mixed intervention design; injectable agents were evaluated together with arthrocentesis, precluding isolation of the standalone effect of TMJ intra-articular injections)
Is injectable platelet rich fibrin beneficial in managing intracapsular temporomandibular disorders? A systematic review and meta-analysis	Bahgat et al., 2025 [26]	Ineligible intervention (mixed intervention design; the independent effect of i-PRF could not be isolated)
Transforming TMJ pain relief: Hyaluronic acid’s efficacy in focus—a comprehensive systematic review and meta-analysis of randomized controlled trials	Flores-Fraile et al., 2025 [27]	Ineligible intervention (included studies combined injections with arthrocentesis, precluding isolation of the standalone effect of TMJ intra-articular injections)
Clinical Efficacy of Prolotherapy for Temporomandibular Joint Disorders: A Systematic Review and Meta-Analysis	Saramantos et al., 2025 [28]	Ineligible intervention (multimodal intervention designs, including periarticular injections, did not allow evaluation of standalone TMJ intra-articular injections)
Effectiveness of platelet-rich plasma for treating temporomandibular joint disorders: a systematic review and meta-analysis of randomized controlled trials	Tsai et al., 2025 [29]	Ineligible intervention (mixed intervention design; the review included adjunctive post-arthrocentesis injection studies, so standalone TMJ intra-articular injection effects were not isolated consistently)
Does intra-articular injection of PRP help patients with temporomandibular joint osteoarthritis after joint puncture? a systematic review and meta-analysis of randomized controlled trials	Xu et al., 2025 [30]	Ineligible intervention (injectable agents were used adjunctively with arthrocentesis, precluding isolation of their standalone TMJ intra-articular effect)
Hyaluronic Acid/Platelet-Rich Plasma Mixture Improves Temporomandibular Joint Biomechanics: A Systematic Review	Chęciński et al., 2024 [31]	Ineligible intervention (injections were combined with arthrocentesis and were not evaluated as standalone TMJ intra-articular interventions)
Intra-Articular Local Anesthetics in Temporomandibular Disorders: A Systematic Review and Meta-Analysis	Lubecka et al., 2023 [32]	Ineligible population (included non-eligible clinical contexts, including healthy volunteers and post-arthroscopy/post-arthroplasty settings)
Efficacy and safety of platelet-rich plasma injections for the treatment of osteoarthritis: A systematic review and meta-analysis of randomized controlled trials	Xiong et al., 2023 [33]	Ineligible population (mixed osteoarthritis population across multiple joints; not specific to TMJ disorders)
Comparative effectiveness of hyaluronic acid, platelet-rich plasma, and platelet-rich fibrin in treating temporomandibular disorders: a systematic review and network meta-analysis	Xu et al., 2023 [34]	Ineligible intervention (injections were combined with arthrocentesis and were not evaluated as standalone TMJ intra-articular interventions)
Autologous Stem Cells Transplants in the Treatment of Temporomandibular Joints Disorders: A Systematic Review and Meta-Analysis of Clinical Trials	Chęciński et al., 2022 [35]	Ineligible intervention (stem cell injections were mostly combined with arthrocentesis, and standalone TMJ intra-articular effects could not be isolated)
The Administration of Hyaluronic Acid into the Temporomandibular Joints’ Cavities Increases the Mandible’s Mobility: A Systematic Review and Meta-Analysis	Chęciński et al., 2022 [36]	Ineligible study design (meta-analysis was not based exclusively on RCTs)
Effectiveness of Intra-Articular Injections of Sodium Hyaluronate, Corticosteroids, Platelet-Rich Plasma on Temporomandibular Joint Osteoarthritis: A Systematic Review and Network Meta-Analysis of Randomized Controlled Trials	Xie et al., 2022 [37]	Ineligible intervention (most included studies combined injectable agents with arthrocentesis, precluding isolation of the standalone effect of TMJ intra-articular injections)
Platelet Concentrate Treatments for Temporomandibular Disorders: A Systematic Review and Meta-analysis	Al-Hamed et al., 2021 [38]	Ineligible intervention (included studies used injections adjunctively with arthrocentesis/arthroscopy, precluding isolation of standalone TMJ intra-articular effects)
Efficacy of hypertonic dextrose injection (prolotherapy) in temporomandibular joint dysfunction: a systematic review and meta-analysis	Sit et al., 2021 [39]	Ineligible intervention (included combined intra-articular and extra-articular injection protocols, so standalone TMJ intra-articular effects could not be isolated)
No Effects of Intra-Articular Injections of Sodium Hyaluronate, Corticosteroids, Platelet-Rich Plasma on Temporomandibular Joint Osteoarthritis: A Systematic Review and Network Meta-analysis of Randomized Controlled Trials	Xie et al., 2021 [40]	Ineligible intervention (most included studies combined injections with arthrocentesis, precluding isolation of the standalone effect of TMJ intra-articular injections)
Effect of Platelet-Rich Plasma Injections on Pain Reduction in Patients with Temporomandibular Joint Osteoarthrosis: A Meta-Analysis of Randomized Controlled Trials	Li et al., 2020 [41]	Ineligible intervention (mixed intervention protocols, including injections used adjunctively with arthroscopy, precluded isolation of standalone TMJ intra-articular effects)
The efficacy of dextrose prolotherapy over placebo for temporomandibular joint hypermobility: A systematic review and meta-analysis	Nagori et al., 2018 [42]	Ineligible intervention (the injected solution was administered across intra- and periarticular sites, precluding isolation of a standalone TMJ intra-articular effect)
Hyaluronate sodium treatment for internal derangement of temporomandibular joint: a systematic review based on randomized controlled trials	Li et al., 2011 [43]	Ineligible intervention (included studies used hyaluronate as an adjunct to lavage, arthroscopy, or splint therapy, precluding isolation of standalone TMJ intra-articular effects)
